# Supplementary material for: CSN5i-3 is an orthosteric molecular glue inhibitor of COP9 signalosome
Source: Nature. 2026 Feb 11;652(8112):1375–83. doi: 10.1038/s41586-026-10129-y (PMC13128448; doi:10.1038/s41586-026-10129-y)

nature portfolio

https://doi.org/10.1038/s41586-026-10129-y

Supplementary information

CSN5i-3 is an orthosteric molecular glue inhibitor of COP9 signalosome

In the format provided by the authors and unedited

Figure 4f

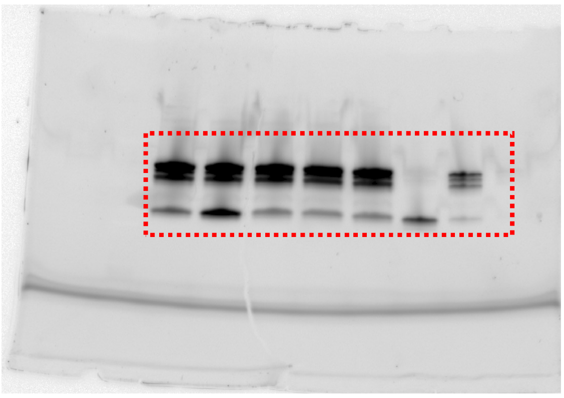

Extended Data Figure 4g

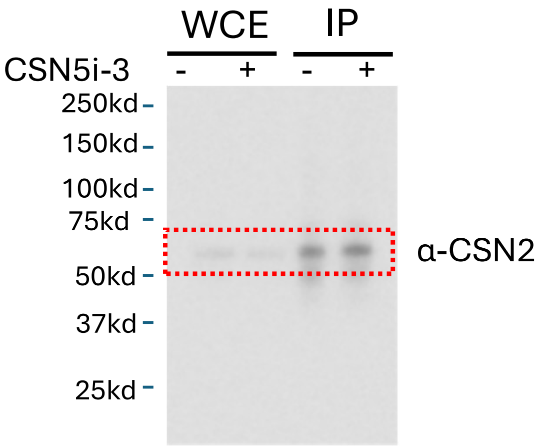

Extended Data Figure 4g

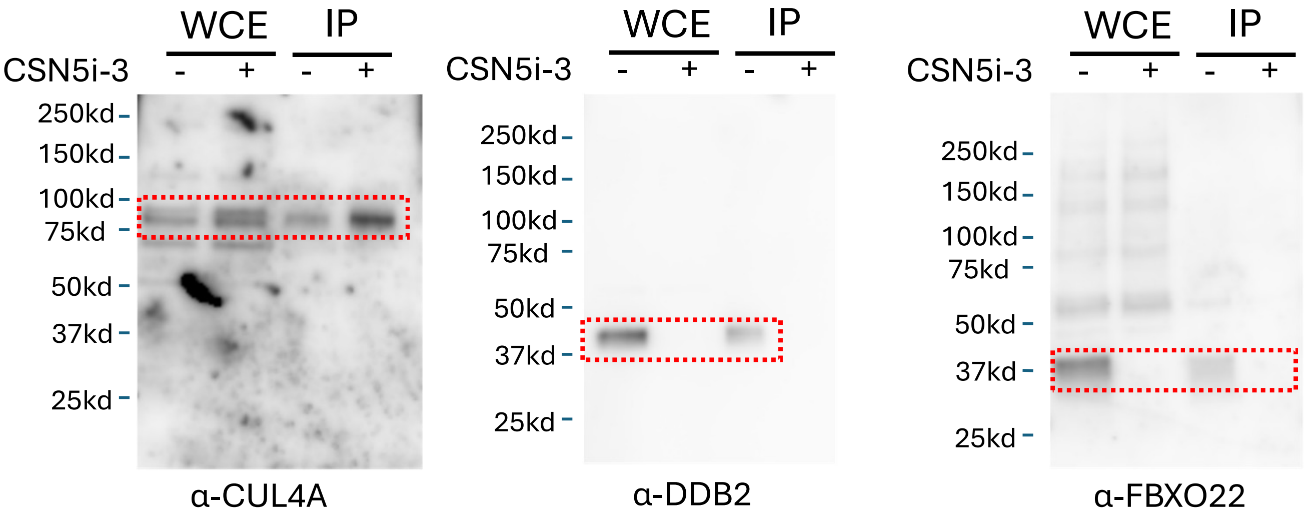

Extended Data Figure 4h

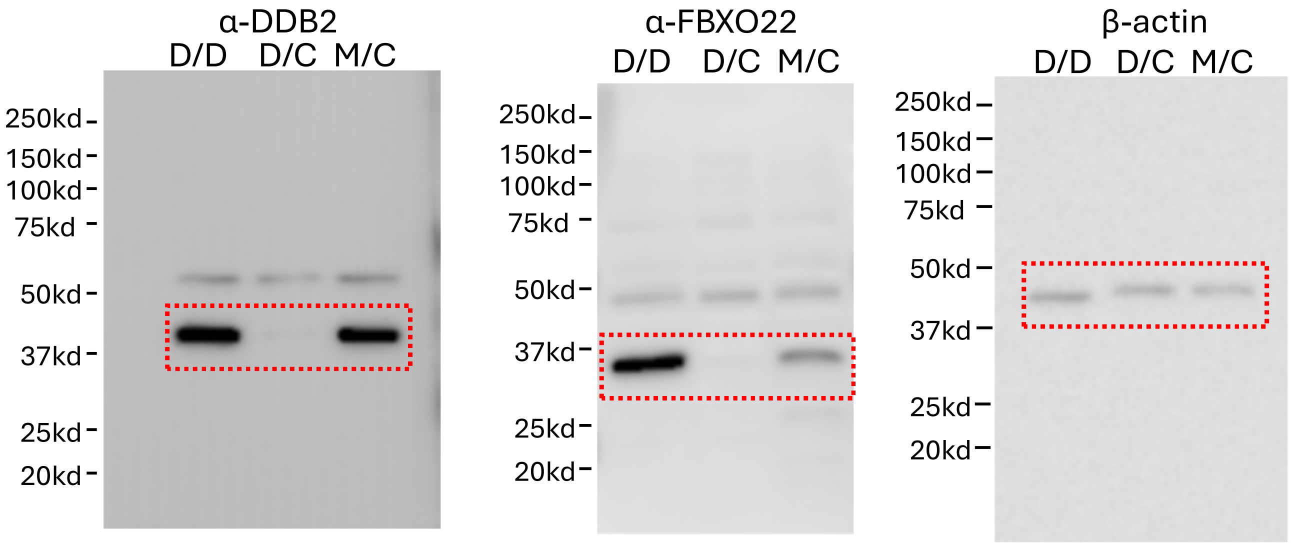

Supplement: Supplementary file 1 — Uncropped gel images. [file 41586_2026_10129_MOESM1_ESM.pdf]
