# Supplementary material for: CSN5i-3 is an orthosteric molecular glue inhibitor of COP9 signalosome
Source: Nature. 2026 Feb 11;652(8112):1375–83. doi: 10.1038/s41586-026-10129-y (PMC13128448; doi:10.1038/s41586-026-10129-y)
Supplement: Supplementary file 2 — Reporting Summary [file 41586_2026_10129_MOESM2_ESM.pdf]

nature portfolio

Corresponding author(s): Lan Huang, Ning Zheng

Last updated by author(s): Nov 25, 2025

Reporting Summary

Nature Portfolio wishes to improve the reproducibility of the work that we publish. This form provides structure for consistency and transparency in reporting. For further information on Nature Portfolio policies, see our Editorial Policies and the Editorial Policy Checklist.

Statistics

For all statistical analyses, confirm that the following items are present in the figure legend, table legend, main text, or Methods section.

n/a	Confirmed	
☐	☒	The exact sample size (*n*) for each experimental group/condition, given as a discrete number and unit of measurement
☐	☒	A statement on whether measurements were taken from distinct samples or whether the same sample was measured repeatedly
☒	☐	The statistical test(s) used AND whether they are one- or two-sided<br>*Only common tests should be described solely by name; describe more complex techniques in the Methods section.*
☒	☐	A description of all covariates tested
☒	☐	A description of any assumptions or corrections, such as tests of normality and adjustment for multiple comparisons
☐	☒	A full description of the statistical parameters including central tendency (e.g. means) or other basic estimates (e.g. regression coefficient) AND variation (e.g. standard deviation) or associated estimates of uncertainty (e.g. confidence intervals)
☒	☐	For null hypothesis testing, the test statistic (e.g. *F*, *t*, *r*) with confidence intervals, effect sizes, degrees of freedom and *P* value noted<br>*Give P values as exact values whenever suitable.*
☒	☐	For Bayesian analysis, information on the choice of priors and Markov chain Monte Carlo settings
☒	☐	For hierarchical and complex designs, identification of the appropriate level for tests and full reporting of outcomes
☒	☐	Estimates of effect sizes (e.g. Cohen's *d*, Pearson's *r*), indicating how they were calculated

Our web collection on statistics for biologists contains articles on many of the points above.

Software and code

Policy information about availability of computer code

Data collection	Cryo-EM: Krios and Glacios Transmission Electron Microscope (Thermo Fisher) with K3 direct electron detector, operated on SerialEM (v4.1).<br>Mass spectrometry data: collected with Orbitrap Fusion Lumos Tune Application (v3.5.3890).<br>BLI data were collected with Octet BLI Discovery (v13.0.3.26).<br>ITC data were collected with MicroCal PEAQ-ITC Control Software (v1.41).
Data analysis	Cryo-EM datasets were processed with cryoSPARC (v4.4.1). The model building used crystal structure model as template, then docked in ChimeraX-1.6 or automatically built with ModelAngelo (v1.0), adjusted with Coot (v0.9.8.91) and refined with PHENIX (v1.20.1-4487-000).<br>Mass spectrometry data were searched using Protein Prospector (v6.3.5; http://prospector.ucsf.edu/prospector/mshome.htm), which was developed by UCSF mass spectrometry facility. Cross-linked peptides were identified through the integration of MS1, MS2 and MS3 data using XL-tools scripts (Wang, et al. Mol Cell Proteomics 2017, 16 (5), 840-854)). MaxQuant (v2.0.3.0) was used for DDA-based label-free quantitation, and DIA-NN (v2.0.2) was used for DIA-based quantitation.<br>BLI data was analyzed with Octet BLI Analysis (v12.2).<br>ITC data were analyzed with MicroCal PEAQ-ITC Analysis Software (v1.41).

For manuscripts utilizing custom algorithms or software that are central to the research but not yet described in published literature, software must be made available to editors and reviewers. We strongly encourage code deposition in a community repository (e.g. GitHub). See the Nature Portfolio guidelines for submitting code & software for further information.

Data

Policy information about availability of data

All manuscripts must include a data availability statement. This statement should provide the following information, where applicable:

- Accession codes, unique identifiers, or web links for publicly available datasets
- A description of any restrictions on data availability
- For clinical datasets or third party data, please ensure that the statement adheres to our policy

The coordinates and cryo-EM maps were deposited in the Protein Data Bank (PDB) and the Electron Microscopy Data Bank (EMDB) with the following accession numbers: CSN: 9E5Z, EMD-47532; CSN5i-3-CSN: 9E81, EMD-47698; CSN5i-1a-CSN: 9PH4, EMD-71639; CSN5i-3-CSN-N8~CRL1: 9EFV, EMD-47981, EMD-47729, EMD-47767; -CSN5i-3-CSN-NEDD8: 9E77, EMD-47660; CSNDM-N8~CRL1: 9EFM, EMD-47976, EMD-47500, EMD-47502; CSNDM-N8~CRL2: 9EFQ, EMD-47977, EMD-47701, EMD-47702; CSNDM-N8~CRL3: 9EGL, EMD-47990, EMD-47776, EMD-47985; CSNDM-N8~CRL4A: 9EG8, EMD-47986, EMD-47543, EMD-47699, and CSNDM-N8~CRL5: 9EG1, EMD-47983, EMD-47663, EMD-47665.
The mass spectrometry data for cross-linking and affinity pulldown [HS15.1]have been deposited to the ProteomeXchange Consortium via the PRIDE partner repository with the dataset identifier PXD063786, PXD063822, respectively.

Research involving human participants, their data, or biological material

Policy information about studies with human participants or human data. See also policy information about sex, gender (identity/presentation), and sexual orientation and race, ethnicity and racism.

Reporting on sex and gender	N/A
Reporting on race, ethnicity, or other socially relevant groupings	N/A
Population characteristics	N/A
Recruitment	N/A
Ethics oversight	N/A

Note that full information on the approval of the study protocol must also be provided in the manuscript.

Field-specific reporting

Please select the one below that is the best fit for your research. If you are not sure, read the appropriate sections before making your selection.

☒ Life sciences ☐ Behavioural & social sciences ☐ Ecological, evolutionary & environmental sciences

For a reference copy of the document with all sections, see nature.com/documents/nr-reporting-summary-flat.pdf

Life sciences study design

All studies must disclose on these points even when the disclosure is negative.

Sample size	No animal or human samples were used in this study. All experiments were performed exclusively with purified recombinant protein complexes, and cell lysates in standard biochemical and structural assays. Therefore, statistical sample size determination is not applicable. For biochemical experiments, we ensured reproducibility by performing each assay in biological replicates (typically n = 3) and confirming key findings by independent replicates. This approach follows established practice for in-vitro enzymology and protein biochemistry where experimental reproducibility—not population sampling—is the relevant metric.
Data exclusions	No data exclusions were performed in this study.
Replication	Where indicated in the paper, experiments were performed in replicate (duplicate or triplicate). Replicate type is specified in the text. All attempts at replication were successful.
Randomization	Randomization is not applicable to this study. All experiments were performed using purified recombinant proteins, and standard cell culture systems used solely for protein expression. These experimental systems do not involve allocation of subjects, biological groups, or treatment arms. Instead, reproducibility was ensured by performing biochemical and biophysical assays in independent replicates, which is the standard practice for mechanistic protein studies.
Blinding	Investigators were not blinded for any of the experiments performed in this study. This was done for practical purposes, and is standard practice for studies employing biochemistry, cell culture, and genomics (Zhang et al., 2018; Vinyard et al., 2019; Haggerty et al., 2021).

Reporting for specific materials, systems and methods

We require information from authors about some types of materials, experimental systems and methods used in many studies. Here, indicate whether each material, system or method listed is relevant to your study. If you are not sure if a list item applies to your research, read the appropriate section before selecting a response.

Materials & experimental systems

n/a	Involved in the study
☐	☒ Antibodies
☐	☒ Eukaryotic cell lines
☒	☐ Palaeontology and archaeology
☒	☐ Animals and other organisms
☒	☐ Clinical data
☒	☐ Dual use research of concern
☒	☐ Plants

Methods

n/a	Involved in the study
☒	☐ ChIP-seq
☒	☐ Flow cytometry
☒	☐ MRI-based neuroimaging

Antibodies

Antibodies used

1) Streptavidin HRP to detect HBTH-CSN2, HBTH-CSN6: Thermo Fisher Scientific, Streptavidin Protein, HRP Cat# PI21126
2) CUL4A antibody: Invitrogen / Thermo Fisher Scientific, Cat# PA5-14542
3) DDB2 antibody: Invitrogen / Thermo Fisher Scientific, Cat# MA5-34832
4) FBXO22 antibody: Proteintech, Cat# 13606-1-AP

Validation

Streptavidin–HRP (Thermo Scientific, Streptavidin Protein, HRP, Cat# 21126) was validated by the manufacturer; application data and supporting citations are provided at: https://www.thermofisher.com/order/catalog/product/21126
CUL4A (Invitrogen / Thermo Fisher Scientific, Cat# PA5-14542) was validated by the manufacturer; application data and supporting citations are provided at: https://www.thermofisher.com/antibody/product/Cullin-4A-Antibody-Polyclonal/PA5-14542
DDB2 (Invitrogen / Thermo Fisher Scientific, Cat# MA5-34832) was validated by the manufacturer; application data and supporting citations are provided at: https://www.thermofisher.com/antibody/product/DDB2-Antibody-clone-JE16-41-Recombinant-Monoclonal/MA5-34832
FBXO22 (Proteintech, 13606-1-AP; RRID: AB_2104403) was validated by the manufacturer; application data and supporting citations are provided at: https://www.ptglab.com/products/FBXO22-Antibody-13606-1-AP.htm

Eukaryotic cell lines

Policy information about cell lines and Sex and Gender in Research

Cell line source(s)

HEK293T was obtained from ATCC, Cat# CRL-3216. HEK293 cell lines stably expressing HBTH-tagged CSN2 or HBTH-tagged CSN6. ExpiSF9 cell line was obtained from Thermo Fisher (A35243).

Authentication

All cell lines were authenticated by Short Tandem Repeat profiling (Genetica). Stable cell lines were also authenticated using affinity purification and western blot analysis.

Mycoplasma contamination

All cell lines tested negative for mycoplasma.

Commonly misidentified lines
(See ICLAC register)

No commonly misidentified cell lines were used.

Plants

Seed stocks	*Report on the source of all seed stocks or other plant material used. If applicable, state the seed stock centre and catalogue number. If plant specimens were collected from the field, describe the collection location, date and sampling procedures.*
Novel plant genotypes	*Describe the methods by which all novel plant genotypes were produced. This includes those generated by transgenic approaches, gene editing, chemical/radiation-based mutagenesis and hybridization. For transgenic lines, describe the transformation method, the number of independent lines analyzed and the generation upon which experiments were performed. For gene-edited lines, describe the editor used, the endogenous sequence targeted for editing, the targeting guide RNA sequence (if applicable) and how the editor was applied.*
Authentication	*Describe any authentication procedures for each seed stock used or novel genotype generated. Describe any experiments used to assess the effect of a mutation and, where applicable, how potential secondary effects (e.g. second site T-DNA insertions, mosiacism, off-target gene editing) were examined.*
